# Supplementary material for: PET imaging-guided chemogenetic silencing reveals a critical role of primate rostromedial caudate in reward evaluation
Source: Nat Commun. 2016 Dec 6;7:13605. doi: 10.1038/ncomms13605 (PMC5150653; doi:10.1038/ncomms13605)
Supplement: Supplementary Information — Supplementary Figures 1-4, Supplementary Table 1 and Supplementary Methods [file ncomms13605-s1.pdf]

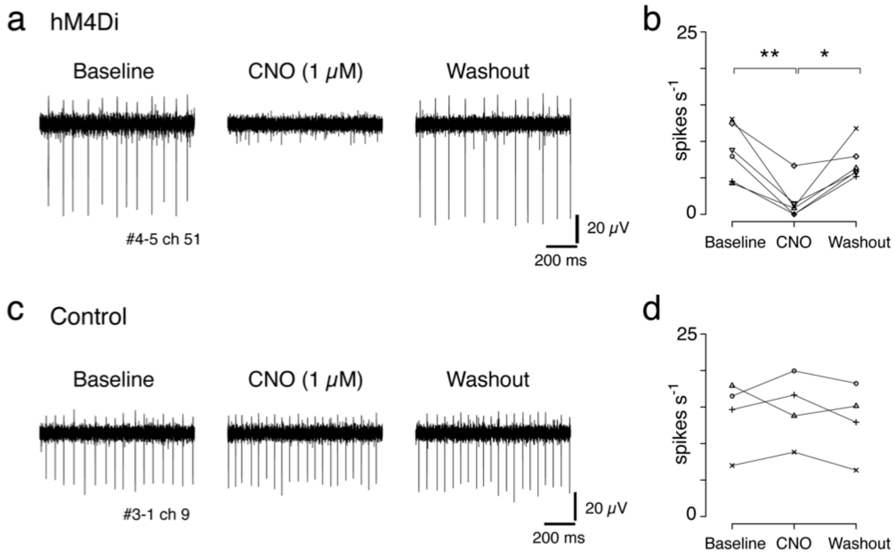

Supplementary Figure 1. CNO-induced silencing in hM4Di-expressed neurons *in vitro*. (**a**) Representative extracellular spike trains from primary cultured neurons prepared from whole brain at 7 days after AAV-hM4Di vector infection before (baseline), 20 min after bath application of CNO solution (CNO, 1μM), and subsequent washout with fresh medium without CNO (washout). (**b**) Firing rate of individual neurons for each condition. The firing rate decreased in the presence of CNO (one-way ANOVA, $F_{2, 10} = 19$, $p < 0.001$; post-hoc pairwise comparison with Bonferroni correction, ** $p < 0.01$, * $p < 0.05$). (**c, d**) Same as a and b, but for non-vector controls, respectively. The firing rate did not change in the presence of CNO (one-way ANOVA, $F_{2, 6} = 0.81$, $p = 0.48$).

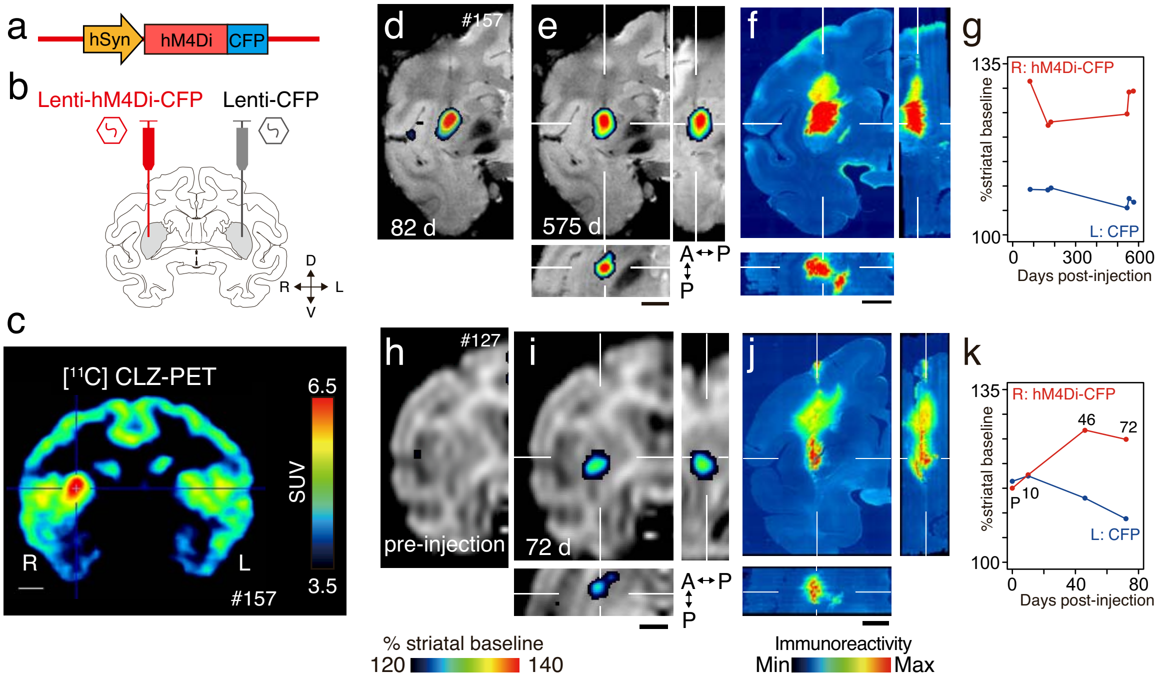

Supplementary Figure 2. Validation of PET visualization for hM4Di-DREADD expression. (**a**) Structure of the lentiviral vector expressing a fusion protein [hM4Di with cyan fluorescent protein (CFP)] under a neuron-specific promoter synapsin (hSyn). (**b**) Illustration of injection locations of the lentiviral vectors. D, dorsal; V, ventral; R, right; L, left. (**c**) A coronal section of [^{11}C]CLZ-PET image representing standardized uptake value (SUV; concentration of radioactivity in the voxel [Bq cm^{-3}] × body weight [g] / injected radioactivity [Bq]) averaged between 30–90 min after injection of radioligand. (**d**) Coronal PET image of focal uptake of [^{11}C]CLZ taken at day 82 after vector injection overlaid on MR images in #157. Uptake values were normalized to the baseline striatal uptake value (% striatal baseline). PET images are shown only for the area over 120% striatal baseline. (**e**) Three projections of focal uptake in [^{11}C]CLZ-PET taken at day 575 after vector injection overlaid on MR images shown in coronal (left), parasagittal (right), and horizontal (bottom) planes showing a needle track for the vector injection. A, anterior; P, posterior; (**f**) Anti-CFP staining of 30 coronal sections taken every 300 µm reconstructed into 3 planes to match panel (e). Relative density of staining is color-coded (see scale). (**g**) Normalized uptake value as a function of days after vector injection in #157. Red and blue dots indicate uptake values at the site of injection of hM4Di-vector and the contralateral control site, respectively. (**h-k**) same as (d-g) except for #127. Pre-injection values are plotted at day 0. Please see **Validation of PET visualization for hM4Di-DREADD expression** section in Methods. Scale bar = 5 mm.

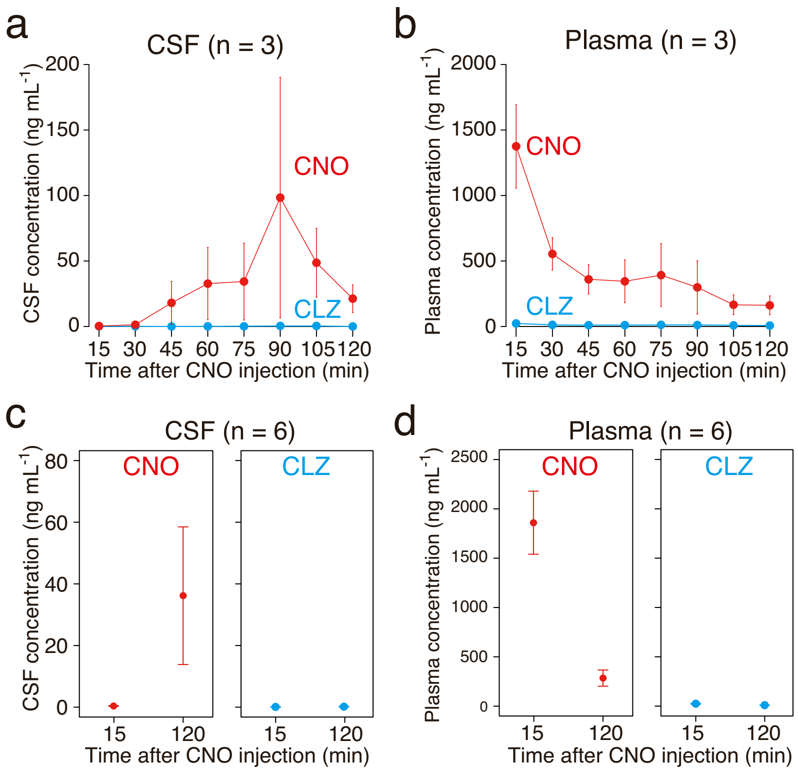

Supplementary Figure 3. Plasma/CSF concentration of CNO/CLZ. (**a and b**) Time course of plasma and CSF concentration (mean ± s.e.m.) of CNO (red) and CLZ (cyan) after administration of CNO (3 mg kg^{-1}, *i.v.*) in three monkeys, respectively. (**c and d**) Plasma and CSF concentration (mean ± s.e.m.) of CNO (left) and CLZ (right) at 15 and 120 min after CNO administration in 6 monkeys, respectively.

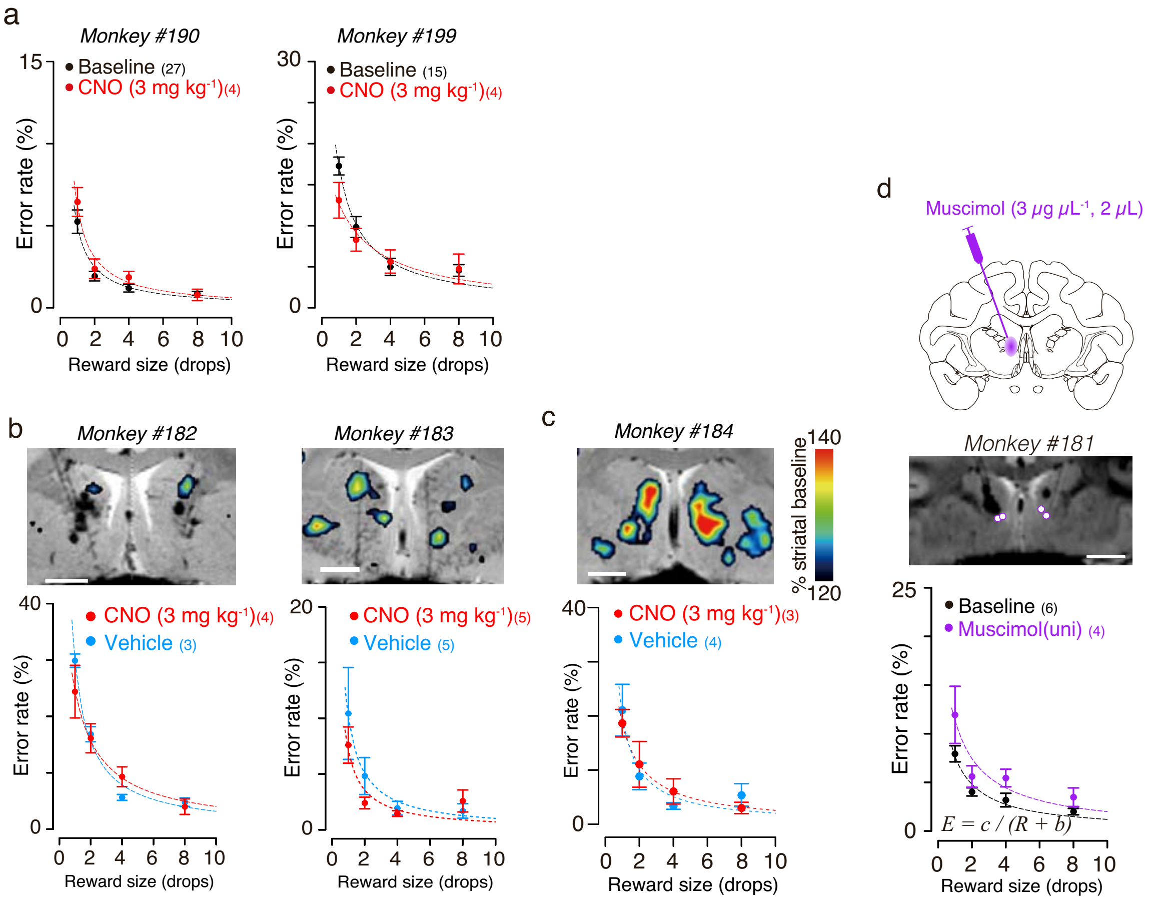

Supplementary Figure 4. Absence of behavioral effect of CNO in monkeys with no/weak or mislocalized DREADD expression, and unilateral chemical inactivation. Error rate (mean ± s.e.m.) as a function of reward size is plotted for CNO treatment (red), baseline (black), vehicle control sessions (blue), and unilateral muscimol inactivation (purple) are shown. (**a**) Effect of CNO treatment on performance in non-vector control in monkeys #190 (before AAV injection) and #199. CNO treatment alone had no effect on error rates (two-way ANOVA, main effect of treatment, $F_{1,30}$ = 1.07, p = 0.30, #190; $F_{1,17}$ = 0.29, p = 0.59, #199). (**b**) *In vivo* hM4Di expression (top) and behavioral results (bottom) in monkeys that received AAV-HA-hM4Di injections. Coronal images of [^{11}C]CLZ-PET overlaid on structural MR image, taken at days 29 and 38 after vector injection are shown for #182 and #183, respectively. CNO treatment had no effect on error rates (two-way ANOVA, main effect of treatment, $F_{1,5}$ = 0.09, p = 0.78, #182; $F_{1,7}$ = 0.27, p = 0.62, #183). (**c**) *In vivo* hM4Di expression (top) and behavioral results (bottom) in a monkey with mistargeted DREADD expression (#184). Coronal image of [^{11}C]CLZ-PET overlaid on structural MR image, taken at day 31 after 1st AAV-hM4Di vector injection. (**d**) Unilateral muscimol inactivation in monkey #181. Illustration of unilateral injection (upper). Location of muscimol injection indicated by open circles (center). Behavioral performance (bottom). Dotted curve is the best fit of inverse function. Scale bar = 5 mm.

Supplementary Table 1. Summary of viral vector injections.

Monkey	Species	Injected vector	Volume (μL)	Titer (particles/mL)	Location
171	Japanese	AAV2-CMV-hM4Di	3 per side	2.0×10^{13}	rmCD (R, L)
184	Rhesus	AAV2-CMV-hM4Di	3 per side*	2.0×10^{13}	rmCD (R, L)
190	Rhesus	AAV2-CMV-hM4Di	3 per side	2.0×10^{13}	rmCD (R, L)
182	Rhesus	AAV2-CMV-HA-hM4Di	3 per side	1.8×10^{13}	rmCD (R, L)
183	Rhesus	AAV2-CMV-HA-hM4Di	3 per side	1.8×10^{13}	rmCD (R, L)
127	Cynomolgus	Lenti-hSyn-hM4Di-CFP	30	2.0×10^{9}	PUT (R)
		Lenti-hSyn-CFP	30	2.0×10^{9}	PUT (L)
157	Cynomolgus	Lenti-hSyn-hM4Di-CFP	30	2.0×10^{9}	PUT (R)
		Lenti-hSyn-CFP	30	2.0×10^{9}	PUT (L)

*: Monkey received injection twice. PUT, putamen.

Supplementary Methods

Histology and immunostaining. Monkeys #127 and #157 were deeply anesthetized with an overdose of sodium pentobarbital (50 mg kg^{-1}, *i.v.*) and transcardially perfused with saline, followed by 4% formaldehyde in 0.1 M phosphate buffered saline (PBS, pH 7.4). The head was mounted on a stereotaxic frame and the brains were cut into a 12-20 mm thick coronal block that included the injection site. The block was postfixed in the same fresh fixative overnight, equilibrated with 30% sucrose and 0.02% NaN_3 in PBS at 4°C, and then cut serially into 50-µm-thick sections on a freezing microtome. For visualization of immunoreactive signals of CFP, a series of every 6th section was immersed in 1% skim milk for 1 h at room temperature and incubated overnight at 4°C with rabbit anti-GFP monoclonal antibody (1:2,000, Life Technologies) in PBS containing 0.1% Triton X-100 and 1% normal goat serum. The sections were then incubated in the same fresh medium containing biotinylated goat anti-rabbit IgG antibody (1:1,000, Jackson ImmnoResearch) for 2 h at room temperature, followed by avidin-biotin-peroxidase complex (ABC Elite, Vector Laboratories) for 2 h at room temperature. For visulalization of the antigen, the sections were reacted in 0.05 M Tris-HCl buffer (pH 7.6) containing 0.04% diaminobenzidine (DAB), 0.04% $NiCl_2$, and 0.003% H_2O_2. The sections were mounted onto gelatin-coated glass slides, air-dried, and coverslipped. A part of other sections was Nissl-stained with 1% Cresyl violet.

Images of sections were digitally captured using an optical microscope equipped with a high-grade charge-coupled device (CCD) camera (Biorevo, Keyence, Osaka, Japan). A series of images of anti-CFP sections (30 and 28 for #157 and #127, respectively) were aligned manually and saved in TIFF format using Adobe Photoshop (Adobe Systems Inc., San Jose, CA) and then reconstructed into 3D rendering images using PMOD.

Measuring CNO/CLZ concentrations in plasma and CSF. Acute blood and CSF samples were collected from saphenous vein and via acute lumbar puncture using a 23-gauge needle, respectively, under ketamine-xylazine anesthesia. Samples were collected at 15 and 120 min after CNO injection (3 mg kg^{-1}, *i.v.*) from 3 monkeys (including #184 and #190, vector-injected and non-vector control monkeys, respectively), which corresponded to just before and 5 min after behavioral testing, respectively. Three other monkeys (#174, #178 and #183) also received 3 mg kg^{-1} CNO, and blood and CSF samples were collected at 15, 30, 45, 60, 75, 90, 105 and 120 min after the administration. Plasma was separated and samples were stored at -80°C until analyzed.

Samples (50 µL) were diluted in 150 µL of methanol followed by other addition of 50 µL of 50% methanol and 20 µL of Granisetron solution (10 ng mL^{-1}, internal standard). Diluted samples were vortexed briefly, then ultracentrifuged at 10,600g at 4°C for 2 min. 50 µL of supernatant from each sample was filtered and diluted at 1:10 or 1:300 with water. Quantification of CNO and CLZ was performed by multiple reaction monitoring (MRM) using a Shimadzu UHPLC LC-30AD system coupled to a tandem MS AB Sciex Qtrap 6500 system. A Capcell Pak C18 MGIII column (Shiseido; 5 µm, 2.0 × 50 mm) was used for metabolite separation. UHPLC was performed with the following: mobile phase A, 0.1% formic acid in water; mobile phase B, 0.1% formic acid in acetonitrile. Gradient: linear gradient from initial condition (5%) to 20% mobile phase B at 2.5 min, then increasing to 95% and held until 3 min, then returning to initial conditions and held for an additional 2 min for column equilibration. The flow rate was 0.4 mL min^{-1}, and the column temperature was maintained at 40°C. The AB Sciex Qtrap 6500 system was operated in MRM ESI+ mode. The following MRM transitions (Q1/Q3) were used to monitor each compound: CLZ (327.1/270.0), CNO (343.1/192.1) and Granisetron (313.2/138.1). Internal standard-normalized area under the peak (response) from serially diluted authentic standard solution was used to build calibration curve for each compound. The concentration of compounds was determined from the calibration curve multiplied by any dilution factor.

***In vitro* electrophysiology.** Whole brain neurons were dissociated and isolated from embryos of B6J mice (embryonic day 14.5) using 'Dissociation Kit' and 'Neuron Isolation Kit' (Miltenyi Biotec, Auburn, CA, USA; cat. no. 130-094-802 and 130-098-752, respectively). Dissociated neurons were diluted at a density of 1 x 10^6 cells mL^{-1} in a neuron culture medium. A 20-µL aliquot of each cell suspension was plated on a poly-L-lysine (Sigma-Aldrich), and Matrigel (BD, Becton, Dickinson and co.)-coated MED64 Probe Multi-electrode Dish (MED-P515A, Alpha MED Scientific, Osaka, Japan), which consisted of 64 electrodes in an 8 × 8 grid with inter-electrode spacing of 150 µm. The neurons were maintained in culture medium [DMEM/F12 (Invitrogen) supplemented with 10% Knockout Serum Replacement (KSR; Invitrogen), N2 (Invitrogen), B-27 Vitamin A (Invitrogen), 10 ng mL^{-1} BDNF, 10 ng mL^{-1} GDNF, 10 ng mL^{-1} NT-3] and incubated at 37°C in a humidified atmosphere with 5% CO_2. At day 7 of culture, the neurons were infected by AAV-hM4Di vector for 48 h. At day 14 of culture, extracellular action potentials of neurons were recorded by a 64-channel recording system (MED64 system, Alpha MED Science) with a sampling rate of 20 kHz for 60 s. The recordings were performed at baseline condition, then 20 min after addition of CNO (1 µM) into culture medium. The recording was also performed at washout condition; the medium was replaced with fresh

medium without CNO and incubated for 20 min for washout of CNO. Extracellular spikes were determined as events when their amplitude exceeded a pre-specified threshold (30 μV). Single units showing regular spiking (< 25 Hz) at baseline condition were chosen for the analysis. Average firing rates across conditions were examined by repeated-measures one-way ANOVA and post-hoc pairwise comparison with Bonferroni correction.
